# Dynamic Mixed-Reality Patient-Specific Aneurysm Clipping Simulation for Two Cases—A Feasibility Study

**DOI:** 10.1227/ons.0000000000001017

**Published:** 2023-12-06

**Authors:** Fredrick J. Joseph, Miguel Cuba, Michael Murek, Andreas Raabe, David Bervini

**Affiliations:** ‡ Image Guided Therapy, ARTORG Center for Biomedical Engineering Research, University of Bern, Bern, Switzerland;; § Department of Neurosurgery, Inselspital Bern University Hospital and University of Bern, Bern, Switzerland

**Keywords:** Aneurysm clipping, Cerebrovascular surgery, Intracranial aneurysm, Microsurgery, Surgical simulation, Preoperative planning

## Abstract

**BACKGROUND AND OBJECTIVE:**

Intracranial aneurysm (IA) clipping is a complex neurosurgical procedure which demands advanced technology to minimize risks and maximize patient outcomes. This study aims to evaluate the feasibility of training patient-specific microsurgical clipping procedures using a mixed-reality physical neurosurgical simulator for unruptured IA.

**METHODS:**

Two board-certified neurosurgeons were asked to simulate surgery in 2 patient-specific left-side unruptured middle cerebral artery-bifurcation IA models. The study was conducted in the operation theater under realistic conditions using a mixed-reality physical neurosurgical simulator. Time, procedural, and outcome-related information was collected. The participating neurosurgeons were encouraged to attempt all possible clipping strategies, even those deemed suboptimal, reporting the outcome of each strategy. Finally, to evaluate the feasibility and added value of integrating indocyanine green fluorescence angiography (ICG-FA) with the simulator, the ICG-FA videos for each clipping strategy were analyzed and compared with the reported clipping outcomes.

**RESULTS:**

Between 4 and 8, different clipping strategies were applied per aneurysm model; the number of strategies was higher in Patient Model 1 (6.5 ± 1.5) (more complex aneurysm) than in Patient Model 2 (5.0 ± 1.0). The clipping strategies differed between surgeons. At most, 53.5 minutes were necessary to complete each training session, but more than double the time was spent on the more complex aneurysm. Up to 53.8% (Patient Model 1) and 50% (Patient Model 2) of the attempted strategies were discarded by the neurosurgeons during the simulation. Evaluation of aneurysm occlusion through ICG-FA was specific, although sensitivity was poor.

**CONCLUSION:**

The present mixed-reality patient-specific simulator allows testing, anticipating, and discarding different aneurysm microsurgical clipping strategies regardless of the pathology complexity. Specific limitations should be considered regarding ICG-FA aneurysm inspection after clipping.

**ABBREVIATIONS:**
ICG-FA: indocyanine green fluorescence angiography
NA: not applicable
NS1: neurosurgeon 1
NS2: neurosurgeon 2
NS1, NS2: Neurosurgeon 1, 2
OT: operation theater
PM1: patent model 1
PM2: patient model 2.

As endovascular treatment becomes a valid alternative to surgery for intracranial aneurysms, aneurysm clipping surgery is commonly reserved, among others, for aneurysms with a wide neck or complex geometry, increasing the challenge and complexity of the surgical procedure. In this context, an appropriate selection of aneurysm clipping strategies is critical to guarantee low morbidity and good outcomes. Furthermore, despite the efficiency of conventional radiology-based aneurysm clip planning, specific intraoperative challenges may be difficult to predict, especially with complex aneurysm morphologies.

Few virtual reality simulation devices have been shown to partially solve this problem by providing an in-depth, immersive view of the surgical procedure as part of preoperative planning. Several indicators and positive effects of these devices, including reduced total surgical time, reduced number of clipping attempts, and increased suitability of preselected clips, have been systematically reported in prospective and retrospective studies.^1-4^ However, in these studies, the 3-dimensional (3D) models lack haptics and tactile feeling of the pathology, which neurosurgeons acknowledge as an additional feature for clip selection and clipping strategy design.^5-7^ To address this limitation, simulations including 3D-printed aneurysm models have been developed and have reported promising results in preoperative real-patient case planning. Some challenges remain, especially regarding replication complexity, scalability, and accuracy in reproducing mechanical properties, such as wall elasticity and blood fluid mechanics.^8-11^

In this context, we aim to evaluate a previously introduced patient-specific mixed-reality simulator (SurgTrain^™^, SurgeonsLab^®^ AG) regarding the feasibility of its use to reproduce microsurgical workflow, including intraoperative indocyanine green fluorescence angiography (ICG-FA), and its added value for case reviews and preoperative planning of aneurysm clipping microsurgery.^11^ Furthermore, intending to meet surgical planning standards in reproducing the most relevant technical, anatomical, and physiological conditions of aneurysm-clipping procedures, we aim to investigate the limitations of the simulator. We tested different clipping strategies in 2 real patient-specific models of already operated cases and compared the clipping strategies. We explored their success and the potential to address the shortages of other current state-of-art solutions.

## METHODS

### Patient-Specific Models

**FIGURE 1.**
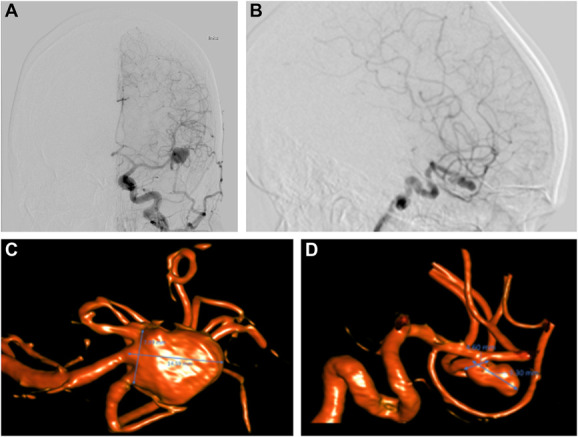
Radiological background of the 2 aneurysm patient models: **A**, planar DSA of PM1, **B**, planar DSA of PM2, **C**, radiological 3D reconstruction of PM1's aneurysm and surrounding arteries, and **D**, radiological 3D reconstruction of PM2's aneurysm and surrounding arteries. 3D, 3-dimensional; DSA, digital subtraction angiography; PM1, Patient Model 1; PM2, Patient Model 2.

Two previously treated aneurysm cases were chosen for the simulation case review (Figure 1). A summary of the pathological background of the cases can be found in Table 1. The 2 cases' 3D-printed true-scale patient-specific replica models used in this study (Patient Model 1 [PM1], Patient Model 2 [PM2]) were developed based on diagnostic radiological image data sets (Figure 1) and consisted of a reduced unilateral craniotomized piece embedding a patient-specific brain model of the temporal and frontal lobes around the Sylvian fissure and a set of connected, hollow, patent high-fidelity artery models replicating the anatomy and nature of the patient's distal internal carotid artery, the M1 and M2 segments of the middle cerebral artery (MCA), and the MCA bifurcation where the aneurysm was located in both patients. The most common pterional approach craniotomy for MCA aneurysms was performed on the training model during fabrication. The internal parts of the skull (ie, skull base structures), the patient-specific brain pieces, and the artery models were designed to preserve the patient's relative anatomical coordinates. The models did not include veins, capillaries, and meningeal tissue, so no dissection but only navigation into the Sylvian fissures with the instruments was required to expose the aneurysm, allowing the models to be reused up to 20 times.

**TABLE 1.** Patient Model Background: MCA Aneurysms

Patient model	Aneurysm type	Aneurysm morphological background		
		Neck size (mm)	Max size (mm)	Neck, dome, aspect ratio
PM1	Left MCA bifurcation	8.0	14.2	1.8
PM2	Left MCA bifurcation	4.6	9.3	2.0

PM1, Patient Model 1; PM2, Patient Model 2; MCA, middle cerebral artery.

### Study Participant Background

Two board-certified cerebrovascular neurosurgeons participated (NS1 and NS2).

### Instruments and Equipment

**FIGURE 2.**
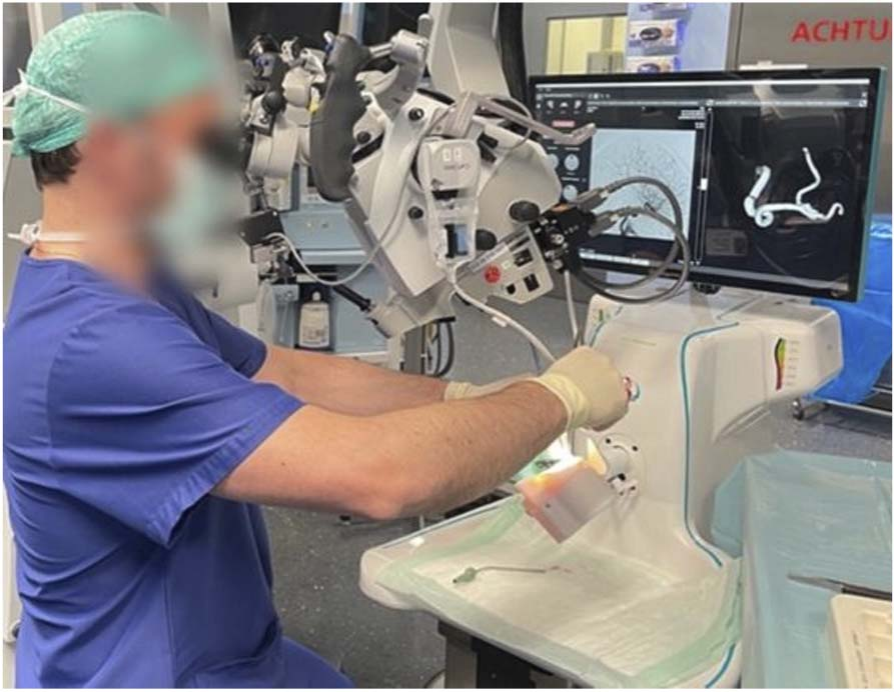
Participant neurosurgeon during a surgical field inspection of model Patient Model 2. The image shows the operation theater background, the surgical microscope, the simulator software with the 3-dimensional segmented aneurysm model and the planar digital subtraction angiography radiological image, the simulator, and the head model.

**FIGURE 3.**
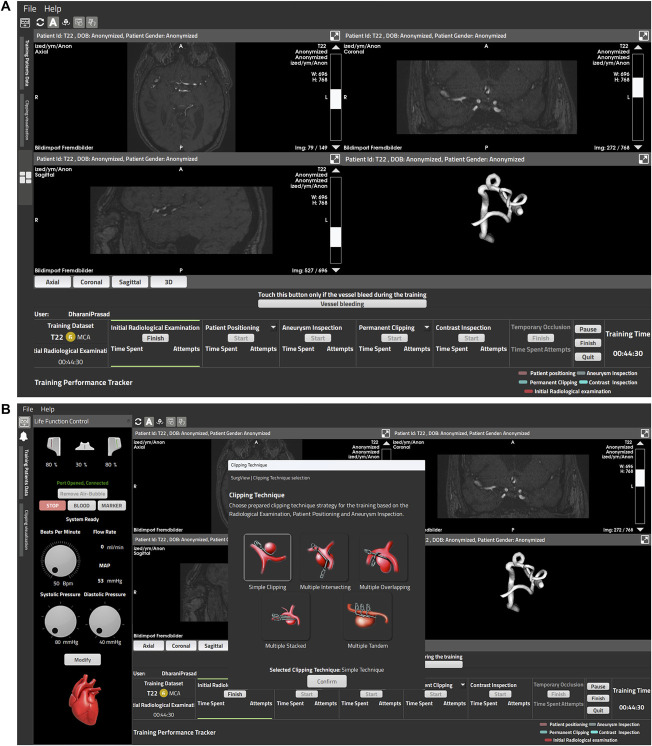
Illustration of the software interface and its main functionalities. **A**, The surgical dashboard, including the radiological visualization module with the axial, coronal, and sagittal views of the patient's preoperative MRI, computed tomography, digital subtraction angiography, and the virtual 3-dimensional rotational reconstruction of the dataset, along with the training performance tracker interface used to record time, approaches, surgical clipping steps, and number of attempts. **B**, On the left, the life function control panel is used to adjust the pumping rate (beats per minute), and systolic/diastolic pressure, and trigger indocyanine green injection and flushing. In the center, the clipping technique selection dialog. Both superimposed onto the surgical dashboard.

The study was conducted on the commercially available simulator SurgTrain^™^ (Figure 2), which provides a portable self-supporting operation theater (OT)–compatible training platform. The simulator is a computerized system consisting of hardware and software elements. The physical elements include a reduced movable and adjustable surgical cart, a joint to hold and rotate the models according to the desired surgical approach, an inbuilt cardiophysiological mimicking pumping unit to perfuse the models with a pulsatile flow of artificial blood, disposable containers to keep nonfluorescent artificial blood separated from the fluorescent artificial blood used for ICG-FA intraoperative evaluation of aneurysm occlusion and vascular patency (ICG VERDYE 5 mg/mL, Diagnostic Green GmbH), an embedded processing and control unit, and a 24-inch 4K touch screen to allow interaction with the software. For its part, a dedicated software interface (SurgView TM, SurgeonsLab AG), accessible alongside the physical components, allows the selection of a patient case, access to their radiological background, and visualization and interaction with the 3D rotational reconstruction, as well as intraoperatively control blood pumping rate, mean arterial pressure, systolic, diastolic pressure, heart rate, blood flowrate, and ICG dye injection and flushing (Figure 3). A neurosurgical microscope (Zeiss Kinevo 900, Carl Zeiss) was used with the simulator in the OT. To perform an aneurysm clipping simulation, standard aneurysm clip trays and instruments from Peter Lazic GmbH and Aesculap AG were provided to the surgeons with complete freedom of choice.

### Study Description

**FIGURE 4.**
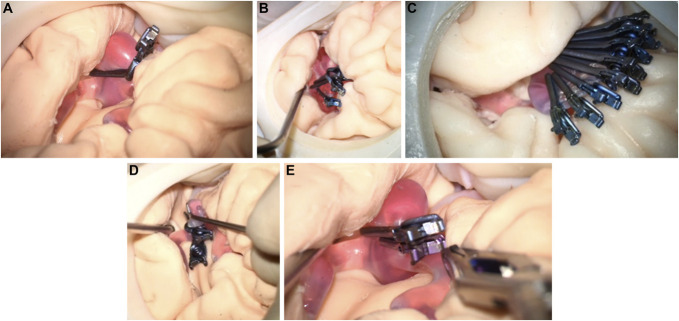
Different clipping techniques used in the study. **A**, simple clipping, **B**, multiple intersecting, **C**, multiple stacked, **D**, multiple overlapping, and **E**, multiple tandem.

The surgical simulation training sessions were video-recorded using cameras with a wide-range perspective. Furthermore, the simulated surgical procedure details were recorded using the neurosurgical microscope camera. The study used 1 aneurysm model in each simulation session, consisting of 2 simulated clipping sessions. The participants used dedicated simulator software to study the patient's background. The experiments included all significant microsurgical steps in aneurysm clipping surgery except craniotomy, durotomy, and arachnoid dissections because the model did not have the complete skull or meningeal tissues. Nevertheless, as a critical and nontrivial step potentially determining the clipping outcomes, the task of finding an optimal orientation of the head model to facilitate the view and the access to the lesion was assigned to the surgeons and explicitly stipulated as part of the complete case-simulation training. The participants were encouraged to expose and clip the corresponding aneurysm model using as many different clip configurations as they could conceive without any time or other procedural constraints, including some configurations not necessarily expected to be successful. Each clip configuration chosen by the participants was classified into several categories called clipping techniques, depending on the quantity and spatial arrangement of the clips. Figure 4 illustrates the different clipping techniques used by the participants. A new clipping strategy was defined as a new approach resulting from changing the clipping technique or the total number and type of clips used. However, minor changes in the selected clip length or slight adjustments in the position and orientation of clips were considered different clipping attempts within the same clipping strategy. The participants repeated the surgical field inspection to evaluate each specific clipping strategy's success visually and labeled the strategy as completed or discarded. Finally, ICG dye was injected into the flow using the simulator software, and intraoperative ICG-FA was performed.

During simulation training, the following procedure-related data were collected in a dedicated database (REDCap 12.5.2, Vanderbilt University).

#### Clipping-Related Data

The count and name of the different strategies applied on each patient model and the number of clipping attempts, ICG-FA inspection events, and temporary and permanent clips used per strategy were recorded.

#### Time-Related Data

The time spent on each of the surgical simulation-related steps was recorded, including the time for radiological examination, head model positioning, aneurysm and surgical field inspection, clipping, and ICG-FA evaluation (if performed). The time needed for temporary proximal occlusion (M1 segment of the MCA artery) was recorded as part of the clipping time when applicable. The time spent on preparation-related aspects, like the time needed to plug in and perfuse the head model, handle the microscope, remove previous clips from models while switching from 1 technique to another, manage a rupture event, and the waiting time after each ICG inspection, was calculated as a single unit by subtracting the time for all the other tasks from the total operating time per session (per aneurysm model).

#### Outcome-Related Data

**FIGURE 5.**
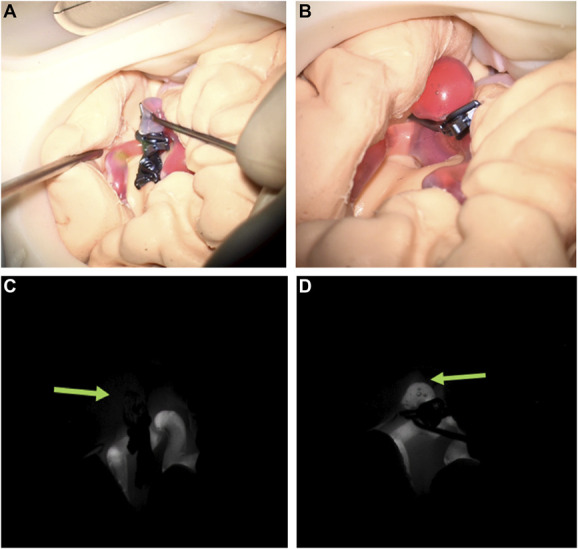
Comparison of microscopic color and ICG-FA fluorescence images corresponding to completed clipping strategies. **A**, Color image of PM2 successfully clipped using an intersecting technique, **B**, color image of PM2 successfully clipped using a single clip (simple clipping), **C**, fluorescent angiographic image of PM2 clipped as shown in **A** (green arrow indicates the location of the aneurysm), where ICG-FA shows agreement with the reported outcome, **D**, fluorescent angiographic image of PM2, clipped as in **B**, where intraoperative ICG-FA shows no agreement with reported success. ICG-FA, indocyanine green fluorescence angiography; PM2, Patient Model 2.

Complications, such as aneurysm rupture or incidental neighboring artery stenosis/occlusion, were recorded. Strategies were labeled as completed or discarded. Later, after the simulation training, the anonymized ICG-FA videos were blindly reviewed by members of the Image-Guided Therapy research group at the University of Bern. The outcomes were labeled as complete or incomplete occlusion according to the presence or absence of indocyanine green dye in the aneurysm sac and compared with the actual clipping outcomes. Figure 5 illustrates this comparison.

### Ethical Disclosure

The state Ethics Committee approved the method of this study and the use of the patient data sets. No patient consent was required.

## RESULTS

The aggregate values for quantitative procedure-related information are presented in Table 2. The participants applied between 4 and 8 different strategies per aneurysm model. The average number of strategies used was slightly higher in PM1 (6.5 ± 1.5) (more complex morphology) than in PM2 (5.0 ± 1.0) and dependent on the neurosurgeon (4.5 ± 0.5 for NS1, 7 ± 1 for NS2). At least 21 minutes and no more than 53.5 minutes were necessary to complete 1 training session, including the different strategies per aneurysm model. Two-fold to 3-fold more time was spent on PM1 than on PM2. Slight differences between surgeons were also observed; NS2 spent 30.5% and 31% more time than NS1 on PM1 and PM2, respectively. Regarding the surgical workflow, the task that required the most time was clipping (53.4% ± 7.8% of total OT time), followed by after-clipping inspection and ICG evaluation (21.7% ± 6.5% of total OT time). Also relevant is the time spent on preparation-related aspects, which was shorter (15.2% ± 2.3% of total OT time).

**TABLE 2.** Summary of Simulation-Aggregated Quantitative Procedure-related Data

Simulation procedure-related data	PM1		PM2	
	NS1	NS2	NS1	NS2
No. of clipping strategies	5	8	4	6
No. of clipping attempts per strategy (mean SD])	1.8 (0.7)	1.5 (0.5)	1.3 (0.4)	1.3 (0.5)
No. of permanent clips per strategy (mean [SD])	2.8 (1.0)	2.5 (3.3)	1.5 (0.5)	1,2 (0.4)
No. of temporary clips per strategy (mean [SD])	1.0 (0.0)	0.9 (1.7)	0 (0)	0.2 (0.4)
Total training time (min)	42	53.5	21.0	27.5
Total time for radiological preparation, head model positioning, and surgical field inspection (min)	3.5	8.0	2.5	1.0
Total time for proximal temporary occlusion of M1 (if any) (min)	3.0	3.0	—	1.0
Total clipping time (min)	25.5	31.0	8.5	15
Total time spent after clipping for aneurysm inspection and ICG-FA evaluation (min)	7.5	7.0	6	7.5

ICG-FA, indocyanine green fluorescence angiography; NS1, Neurosurgeon 1; NS2, Neurosurgeon 2; PM1, Patient Model 1; PM2, Patient Model 2.

Preparation-related time data are not reported in this table.

Table 3 contains descriptive information about the applied clipping strategies and their outcomes. In this regard, differences were observed between participants considering the use of clipping techniques. NS1 applied the multiple tandem clipping techniques in most cases (55.6%), followed by the simple technique, while NS2 used the simple technique most often (57.1%), followed by the multiple tandem clipping techniques. For PM1, 46.2% of the strategies were completed successfully, whereas 53.8% were discarded by the neurosurgeons and not completed. For PM2, half of the strategies were completed, and the other half were discarded.

**TABLE 3.** Summary of Clipping Strategies and Techniques and Comparison of Actual and Measured Outcomes

Patient model	Participant	Clipping technique	Strategy ID	No. clipping attempts	Rupture	Outcome	Aneurysm occlusion based on ICG-FA inspection
PM1	NS1	Simple	s1	1	No	Discarded	NA
		Multiple tandem	s2	1	No	Complete	Incomplete
			s3	1	No	Discarded	NA
			s4	2	No	Complete	Incomplete
			s5	2	No	Complete	Incomplete
	NS2	Simple	s1	2	No	Discarded	NA
			s4	1	No	Discarded	NA
			s7	2	No	Discarded	NA
		Multiple stacked	s3	2	No	Complete	Complete
		Multiple tandem	s2	2	No	Complete	Incomplete
			s5	1	No	Discarded	NA
			s8	1	No	Complete	Incomplete
		Other	s6	1	Yes	Discarded	NA
	Rate of completion/success					46.2%	
PM2	NS1	Simple	s3	2	No	Discarded	NA
			s4	1	No	Discarded	NA
		Multiple tandem	s1	1	No	Complete	Incomplete
		Multiple overlapping	s2	1	No	Complete	Complete
	NS2	Simple	s1	1	No	Discarded	NA
			s2	2	No	Complete	Incomplete
			s3	2	No	Complete	Complete
			s4	1	No	Complete	Incomplete
			s5	1	No	Discarded	NA
		Multiple stacked	s6	1	No	Discarded	NA
	Rate of completion/success					50%	

ICG-FA, indocyanine green fluorescence angiography; NA, not applicable; NS1, Neurosurgeon 1; NS2, Neurosurgeon 2; PM1, Patient Model 1; PM2, Patient Model 2.

An evaluation of the integration of ICG-FA technology in the simulation is illustrated in more detail in Table 4, using the actual clipping outcomes reported by the surgeons as a gold standard. The method showed poor sensitivity (27%) in many cases where ICG-FA failed to be consistent with the reported strategy outcome.

**TABLE 4.** Sensitivity of ICG-FA as a Method to Measure Aneurysm Occlusion

	Complete (ICG-FA)	Incomplete (ICG-FA)	Sensitivity
Complete (reported)	3	8	27.3%

ICG-FA, indocyanine green fluorescence angiography.

## DISCUSSION

The high number of clipping strategies that the neurosurgeons were able to test during the simulation sessions (4 to 8 different strategies per patient model), the observed surgeon-to-surgeon variability in the use of clipping techniques, and the fact that they achieved complete occlusion of the same aneurysms through different techniques reflect that the simulation is not restrictive to 1 or even to a predetermined set of surgical approaches and, while it is always solvable, does not converge to a single solution but provides neurosurgeons with a tool to train and test different clipping strategies at their discretion. This makes the simulation robust and adaptive to the surgeon's preferences and specific skills. Moreover, the simulator allowed the participants to discard multiple strategies before coming to an optimal clipping solution, which highlights its utility in surgical planning contexts, where the aim is not to train or rehearse basic standard procedures but to recreate the conditions of a real surgery to anticipate suitable strategies, discard unsuccessful approaches that could have failed in real surgery, and even test seemingly suboptimal approaches which for safety reasons would have never been tested on a real patient. This has the potential to create a complete autonomous learning experience even for senior neurosurgeons at a low cost because the models are reusable, and there exists no risk of damage to the patient.

Less than 1 hour of simulation time was necessary to test all the techniques. This suggests that the simulation can represent an adjunct step to conventional radiological planning that allows extensive rehearsal of different techniques specific to a single patient. Differences were observed between the 2 models when considering the same surgeon, such as differences in time spent on the clipping sessions and in the total number of strategies applied. The longer operating times and the more significant number of applied strategies, in contrast with the fewer completed attempts and lower clipping success rate recorded for PM1, are consistent with our prior assumption regarding the higher level of complexity and aneurysm morphology in PM1. These results reflect the high level of anatomical fidelity, which is a good indicator of a successful recreation of the actual surgical conditions and is crucial when envisioning the simulator as a future patient-specific surgical planning tool.

In addition, the low variance of the time for preparation-related aspects across the clipping sessions suggests that the simulation is robust and that contingencies are either infrequent or can be managed quickly so that they do not significantly affect the duration of the sessions.

### Limitations

The major limitation found was a significant reduction in the occluding effect of the clips leading to the low sensitivity of ICG-FA evaluation compared with the clipping outcomes reported by the neurosurgeons. Some potential causes have been identified for this phenomenon. First, the wall thickness of the aneurysm models is suspected to be higher than typical metrological values, especially in the dome and neck area. In this situation, when a clip is applied, the thick walls push the blades apart, impeding complete occlusion of the aneurysm. Another possible coexisting phenomenon is that the closing force of the clips deteriorates over time with continued reuse, which the manufacturers have acknowledged. Any of the latter would prevent the aneurysm from being properly occluded even if the clipping technique was correct, thus letting the indocyanine green dye flow through the aneurysm neck during the ICG-FA evaluation phase.

Other than this, it must be noted that the craniotomy procedure, microsurgical dissection aspects, the surgical access creation, and exposure of the microneuroanatomical structures other than the main MCA bifurcations were not included in the training head model at the time of this study; in the future, they can help prepare the surgeons in conditions more like those of an actual patient.

Because this study was a feasibility study, a more extensive variety of patient models with different types of aneurysms and more participant neurosurgeons with varying experience levels would help reinforce the findings and conclusions. In addition, a comprehensive collection of data with a larger cohort would allow to compare clip patterns and strategies among surgeons and to perform more detailed analysis to support specific dependencies between variables suggested by the results obtained in this study. Finally, future researchers should assess direct skill transfers to real-life clinical settings and preoperatively, to determine whether the simulator improves single-patient outcomes, increases patient safety, reduces costs, and improves surgeons' confidence.

## CONCLUSION

The dynamic mixed-reality patient-specific aneurysm clipping simulator and training models recreate the conditions of intracranial aneurysm clipping and allow neurosurgeons to test and train multiple clipping strategies and personalized treatment approaches. In addition, the simulation can be completed in a reasonable time, facilitating its incorporation into standard preoperative surgical planning protocols. Specific limitations were identified regarding ICG-FA use. These findings illustrate a path for further developments in aneurysm clipping simulation technology and encourage future research to identify whether these solutions could be introduced into clinical practice to gain long-term benefits.

## COMMENTS

The authors describe the use of a 3D-printed model of real patients' anatomy to simulate the microsurgical clipping of cerebral aneurysms. Their work demonstrates the feasibility of implementing such a model to learn microsurgical aneurysm treatment and also to rehearse and refine clip reconstruction in preparation for uniquely challenging cases.

Simulation has become an important part of training and preparation in other disciplines such as the airline industry and military. Both industries practice with tools such as virtual flight or combat simulators to rehearse high-risk maneuvers in an environment with essentially no stakes.

Cerebrovascular neurosurgery involves risk assessment, and there is considerable interest in simulation to help train future generations of surgeons. Simulators exist in different forms with variable strengths and weaknesses. Digital simulators using virtual or augmented reality allow for repetition and can be designed to simulate specific cases but often lack realism and tactile feedback. As a result, digital simulators are helpful to review anatomy but have a limited utility in the rehearsal of technical skills. In comparison, physical models such as cadavers can be more realistic and allow for physical interaction to teach surgical technique. However, these models are often resource-limited and dependent on the anatomy presented.

The shortcomings of existing simulation tools mean that most surgical education relies on real-world experience under the tutelage of a senior surgeon. Owing to variability in training, there may be limited exposure to techniques that are required in independent practice. In neurovascular neurosurgery with the expansion of endovascular approaches for the treatment of intracranial aneurysm, there are arguably fewer opportunities for the junior neurosurgeon to learn safe and effective technique for microsurgical aneurysm clipping. Sophisticated models such as the 1 presented here offer the potential to help fill this learning gap by allowing trainees to prepare outside of the operating room to maximize their learning from real-world opportunities. Future studies will focus on the significant challenges of 3D model training such as replication of case specific anatomic nuances, exquisite haptic feedback, and large-scale implementation.

**David Bailey and Ephraim W. Church**

*Hershey, Pennsylvania, USA*
